# Optimization of RNAscope for HPV mRNA detection in liquid-based cervical cytology samples

**DOI:** 10.3389/fonc.2026.1766237

**Published:** 2026-03-11

**Authors:** Tianqi Liu, Xianrong Zhou, Xiang Tao, Hao Zhang, Tingting Chen

**Affiliations:** Department of Pathology, Obstetrics & Gynecology Hospital of Fudan University, Shanghai Key Lab of Reproduction and Development, Shanghai Key Lab of Female Reproductive Endocrine Related Diseases, Shanghai, China

**Keywords:** cervical lesion, HPV E6/E7 mRNA, *in situ* Hybridization, liquid-based cytology, RNAscope

## Abstract

**Purpose:**

To establish an RNAscope-based joint detection scheme compatible with liquid-based cytology (LBC) slides and staining systems, enabling simultaneous observation of cytological phenotype and HPV E6/E7 mRNA expression in cervical cancer and precancerous lesions.

**Methods:**

From March 2023 to February 2024, 115 LBC samples were collected at the Affiliated Obstetrics and Gynecology Hospital of Fudan University. Histopathological diagnosis included 30 LSIL, 33 HSIL, 29 HPV-related SCC, 8 HPV-related adenocarcinoma and 15 negative for cervical intraepithelial lesions cases. RNAscope technology, originally applied to paraffin samples, was adapted for LBC by modifying slide type selection, extending target retrieval, and enhancing permeabilization. The improved RNAscope *in situ* hybridization was then employed to detect high-risk HPV E6/E7 mRNA. Positive rates, diagnostic concordance with histopathology and, expression pattern were statistically analyzed.

**Results:**

RNAscope combined with LBC showed high positive rates, 93.33% for LSIL, 96.97% for HSIL, 100.00% for SCC, and 87.50% for HPV-related adenocarcinoma. Compared with HPV PCR testing, the diagnostic concordance rate reached 95.65% (P<0.001). Figures illustrate RNAscope positive expression patterns in LSIL, HSIL, SCC and, HPV-related adenocarcinoma.

**Conclusion:**

The adaptation of RNAscope technology, originally designed for paraffin samples, enables accurate diagnosis in cervical LBC, enhances consistency with histopathology, and provides a novel diagnostic approach for cervical lesions.

## 1 Introduction

Cervical cancer is one of the most common malignant tumors of the female reproductive tract in China, with an incidence rate ranking second only to breast cancer. In 2022, there were 150,659 new cases and 55,654 deaths in China (1). Cervical liquid-based cytology (LBC) is the mainstream screening method and plays a crucial role in early diagnosis. However, accurate interpretation of LBC smears remains challenging. Diagnostic accuracy depends heavily on pathologists’ expertise and is further affected by sample preparation, patient age, lesion location, and even pathologist fatigue and emotions (2, 3). Regional differences in diagnostic quality further hinder effective identification of high-risk populations, increasing the risk of missed and incorrect diagnoses. In recent years, LBC has been gradually replaced by high-risk HPV DNA testing as primary screening method in many high-income countries (4–6). While this method has high sensitivity, its specificity is low, and not all women need treatment because low-grade lesions can regress without intervention, often leading to unnecessary colposcopy referrals (7). Moreover, HPV DNA testing only detects viral presence without providing *in situ* information. It may identify transient or past infections that are not transcriptionally active, raising the risk of overtreatment (8).

RNAscope analysis employs an innovative *in situ* hybridization technique to visualize individual RNA molecules within each cell of a sample mounted on a glass slide, even enhancing signals in long-term stored or partially degraded samples (9). It enables *in situ* detection of HPV E6/E7 mRNA and has been widely applied in FFPE samples of cervical cancer and head and neck tumors (10–12). This method allows direct microscopic observation of HPV E6/E7 transcriptional activity and has shown high sensitivity and specificity for cervical cancer (11, 13). However, to date, the application of RNAscope in LBC preparations has only been documented in head and neck squamous cell carcinoma, with no reports in cervical lesions (14). The weak adhesion of cervical exfoliated cells also presents a potential challenge for its adaptation to LBC samples. To adress the limitations of cytology-based diagnosis and follow up HPV viral status, we improved and validated an RNAscope *in situ* hybridization method compatible with LBC slides and staining systems. This method is designed for the triage of equivocal cytology, as well as for the precise diagnosis and differential diagnosis of challenging HPV-related cervical lesions.

## 2 Materials and methods

### 2.1 Case selection

From March 2023 to February 2024, 115 LBC samples were collected from patients with histopathologically confirmed cervical intraepithelial lesions, squamous cell carcinoma (SCC), or adenocarcinoma at Fudan University Affiliated Obstetrics and Gynecology Hospital. The cohort included 30 cases of low-grade intraepithelial lesions (LSIL), 33 cases of high-grade intraepithelial lesions (HSIL), 29 cases of HPV-related SCC, 8 cases of HPV-related adenocarcinomas and 15 cases of negative for cervical intraepithelial lesions. Patients were included if they 1) underwent both LBC and HPV genotyping tests, 2) had a cervical biopsy performed in our hospital with definitive pathological results, and 3) successfully completed *in situ* hybridization detection. Patients were excluded if they 1) had incomplete clinical and laboratory data and 2) tissue sections with missing or indeterminant lesions. Histopathological results served as the gold standard. A total of 15 negative cases were randomly selected from the large negative sample pool using a computer-generated random number table. Owing to the relatively low incidence of HPV-related adenocarcinomas, the sample size in this study is inherently limited. However, to mitigate selection bias and ensure the best possible representation of the disease spectrum, we consecutively enrolled all eligible patients who met the inclusion criteria during the study period. Remaining LBC specimens were subjected to RNAscope testing and RNAscope interpretation was carried out independently by two trained researchers who were blinded to the cytological and histopathological results. Final assessments were determined by consensus between the two evaluators. Additional pathological data were retrieved from the department archives.

### 2.2 LBC examination

Cytological preparations were made using the BD SurePath system (BD Diagnostics, Franklin Lakes, NJ). After separating the brush head from the handle, it was placed into a SurePath^®^ sample storage solution. The sample was vortexed, transferred to PrepStain^®^ density separation reagent, and centrifuged. Cell clusters were resuspended, mixed, and transferred to the PrepStain settling chamber, where cells settle by gravity. Smears were then stained and sealed for routine cytological screening and classification. Cytological diagnoses were made according to the 2014 Bethesda System terminology for cervical or vaginal cytology (15). The SurePath^®^PreCoat slide provided well-preserved, evenly stained cell populations within a 13mm circle area. This method largely eliminated air-drying artifacts, overlapping fragments, and excessive white blood cells, thereby improving visualization of epithelial cells, diagnostic cells, and infectious organisms (16).

### 2.3 HPV genotyping testing

A cervical sampling brush was inserted into the squamocolumnar junction to collect exfoliated cells, which were placed in a sampling tube labeled with the patient number and sealed for testing. The HPV genotyping testing was performed by BioPerfectus Multiplex Real-Time PCR (BMRT) assay (BioPerfectus Technology Co, Taizhou, China), including high-risk types (HPV 16, 18, 26, 31, 33, 35, 39, 45, 51, 52, 53, 56, 58, 59, 66, 68, 73, and 82) and low-risk types (HPV 6, 11, and 81).

### 2.4 RNAscope *in situ* hybridization detection

The RNAscope HPV HR18 multi-subtype detection kit (Advanced Cell Diagnostics, USA) was used to detect HPV E6 and E7 mRNA fragments using probes for 18 high-risk HPV subtypes (HPV 16, 18, 26, 31, 33, 35, 39, 45, 51, 52, 53, 56, 58, 59, 66, 68, 73, and 82), with a dapB probe as the negative control and a PPIB probe as the positive control. To improve probe penetration, the standard protocol was modified by adding a PBST permeabilization step (1% Triton X-100 in PBS), which dissolves membrane lipids and facilitates nuclear probe entry (17). Briefly, additional LBC smear slides were prepared and dried at 60 °C for 5 minutes, incubated with hydrogen peroxide at room temperature for 10 minutes, and wash with distilled water. Target retrieval was performed for 15 minutes, followed by a distilled water wash, then digestion with gastric protease at 40 °C for 30 min and another wash. After permeabilization with 1% PBST for 15 min and washing, slides were hybridized with the HR18 multi-subtype probe at 40 °C for 2 h. Signal amplication was carried out sequentially with Hybrid Amp1-6 (30, 15, 30, 15, 30, and 15 min, respectively, at either 40 °C or room temperature), with buffer washes between steps. Finally, slides were developed with DAB, rinsed with tap water, counterstained with hematoxylin, dehydrated in gradient ethanol, cleared in xylene, and mounted with neutral resin. Positive signals were observed as distinct brown dot-like particles within cells, with no background non-specific staining.

### 2.5 Statistical processing

Data were analyzed using SPSS version 25.0. Categorical variables were expressed as percentages (%). Intergroup comparisons were performed using the McNemar test, and a value of P<0.05 was considered statistically significant.

## 3 Results

### 3.1 Optimization of RNAscope in liquid-based cervical cytology samples

#### 3.1.1 Cell smear pretreatment and comparison of slide types

**Figure 1:**
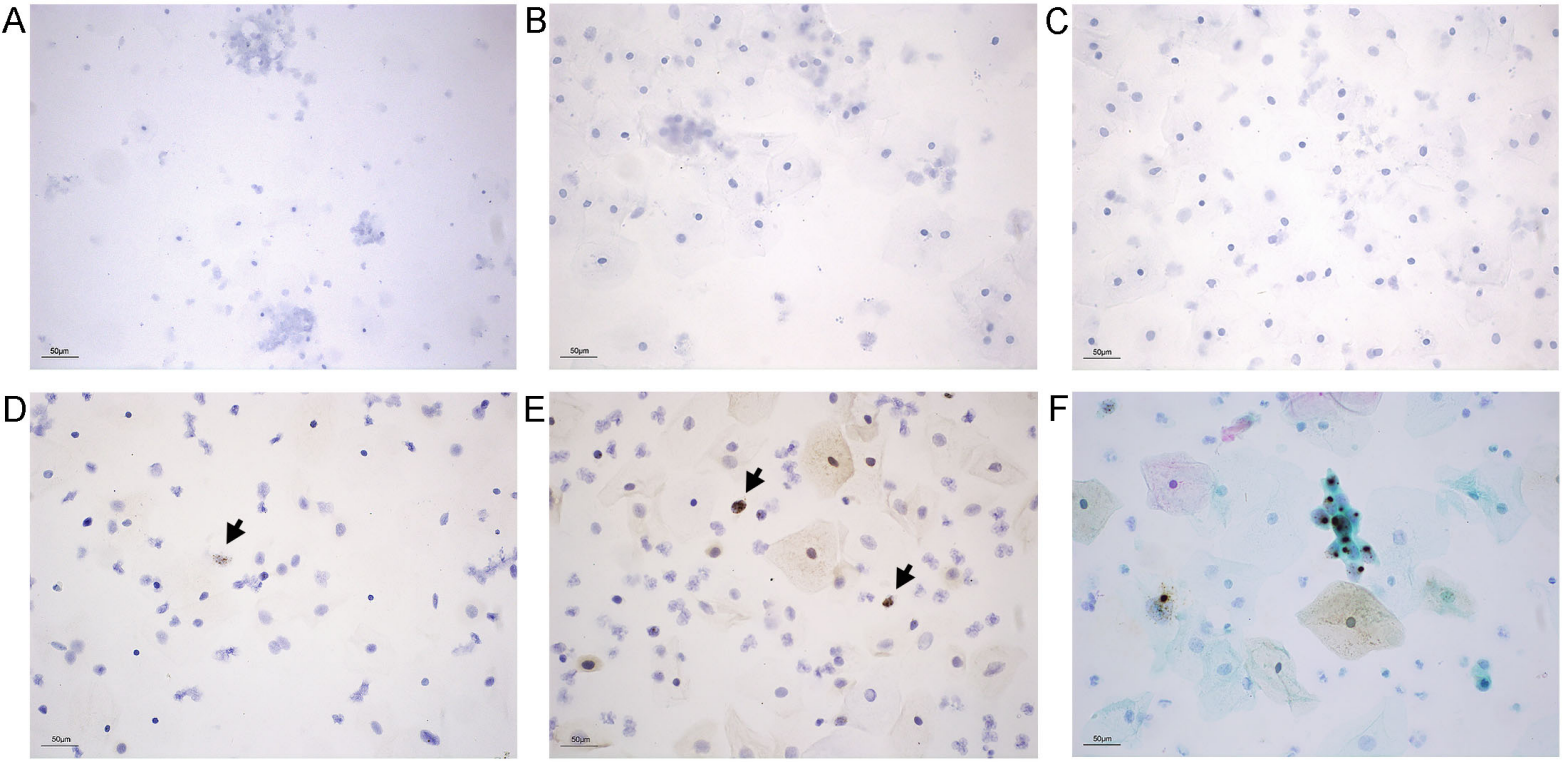
Improvement of the RNAscope method in cervical LBC samples. **(A–C)** Distribution of normal cervical cells on different slides: **(A)** plain slide, **(B)** adhesion slide, and **(C)** SurePath^®^PreCoat slide **(D, E)** Comparison of positive RNAscope signal in cervical squamous cell carcinoma samples under different retrieval conditions (positive signals shown by black arrows): **(D)** target retrieval for 8 min and **(E)** target retrieval for 15 min with PBST permeabilization. **(F)** Liquid-based smear of cervical squamous cell carcinoma after Pap staining. Scale bar, 50 μm (applies to all panels).

Samples without intraepithelial neoplasia were used for pretreatment experiments. Undried smears showed severe cell detachment, whereas smears dried at 60 °C for 5 min remained intact cells. All plain glass slides, adhesion glass slides and SurePath^®^PreCoat slides are tested for cell smear preparation. Cells adhered in clusters on both plain and adhesion slides, but SurePath slides provided uniform cell distribution within a 13mm diameter and effectively prevented detachment during target retrieval (**Figures 1A–C**).

#### 3.1.2 Optimization of target retrieval and PBST permeabilization

To enhance positive signal intensity in liquid-based smears, target retrieval time was extended, and a PBST permeabilization step was added. At 8 min retrieval, only a few cells showed weak positive signals. Extending retrieval to 15 min combined with 1% PBST permeabilization (15 min at room temperature) produced clear brown dot-like signals across all fields, with markedly improved intensity and abundance. Signals were easily visible even under low magnification (10x) (**Figures 1D, E**).

#### 3.1.3 Papanicolaou staining overlay

To distinguish basal, intermediate, and superficial cells, papanicolaou staining was applied after hematoxylin counterstaining. This overlay improved cytomorphological interpretation and supported precise diagnosis in cervical LBC (**Figure 1F**).

### 3.2 Comparative application and interpretation of RNAscope in liquid-based cervical cytology samples

#### 3.2.1 Comparison of detection methods

This study included 115 cervical LBC samples from patients with a median age of 45 years (range: 18–79). HPV genotyping revealed that other high-risk types were most common (41 cases, 35.65%), followed by HPV 16/18 only (37 cases, 32.17%) and mixed infections (24 cases, 20.87%). Cytologically, abnormal findings predominated, with HSIL being the most frequent diagnosis (32 cases, 27.83%), followed by SCC (23 cases, 20.00%) and LSIL (20 cases, 17.39%). NILM samples accounted for 18 cases (15.65%). Detailed clinical characteristics are presented in **Table 1**.

**Table 1:** Clinical characteristics of 115 cervical LBC samples.

Characteristic	Subcategory	n (%)
Age, median (range) – yr		45 (18 – 79)
HPV genotype	HPV 16/18 only	37 (32.17)
	Other high-risk types only	41 (35.65)
	Mixed infection (16/18 + other high-risk)	24 (20.87)
Cytology	NILM	18 (15.65)
	ASC-US	6 (5.22)
	ASC-H	8 (6.96)
	LSIL	20 (17.39)
	HSIL	32 (27.83)
	SCC	23 (20.00)
	AGC	4 (3.48)
	AIS	4 (3.48)

Values are n (%) unless otherwise noted. AGC, atypical glandular lesion; AIS, adenocarcinoma *in situ*; ASC-US, atypical squamous cells of undetermined significance; ASC-H, atypical squamous cells cannot exclude HSIL; NILM, negative for intraepithelial lesion or malignancy.

The positive detection rates for HPV genotyping and RNAscope *in situ* hybridization are presented in **Table 2**, where “+” denotes concordance between LBC interpretation and histopathology. Across all lesion types, RNAscope showed a higher positive rate than HPV testing, with an overall diagnostic concordance rate of 95.65% relative to histopathology. A McNemar test confirmed a statistically significant difference between the two detection methods (P<0.001). Using histopathology as the reference standard, the RNAscope test combined with LBC yielded a sensitivity was 96.0% (95% CI: 90.1–98.9%), a specificity of 93.3% (95% CI: 70.2–98.9%), a positive predictive value (PPV) of 99.0% (95% CI: 94.1–99.9%) and a negative predictive value (NPV) of 77.8% (95% CI: 52.4–93.6%), with an overall diagnostic consistency of 95.65% (95% CI: 90.1–98.6%).

**Table 2:** Comparison of positive detection rates between two methods in 115 cases of cervical lesions (n, %).

Histopathology	RNAscope +	HPV DNA +
LSIL (n=30)	28 (93.33)	19 (63.33)
HSIL (n=33)	32 (96.97)	27 (81.82)
HPV-related SCC (n=29)	29 (100.00)	23 (79.31)
HPV-related adenocarcinoma (n=8)	7 (87.50)	4 (50.00)
Negative for cervical intraepithelial lesions (n=15)	14 (93.33)	15 (100.00)
Diagnostic concordance rate	95.65	76.52

#### 3.2.2 Interpretation of RNAscope results

**Figure 2:**
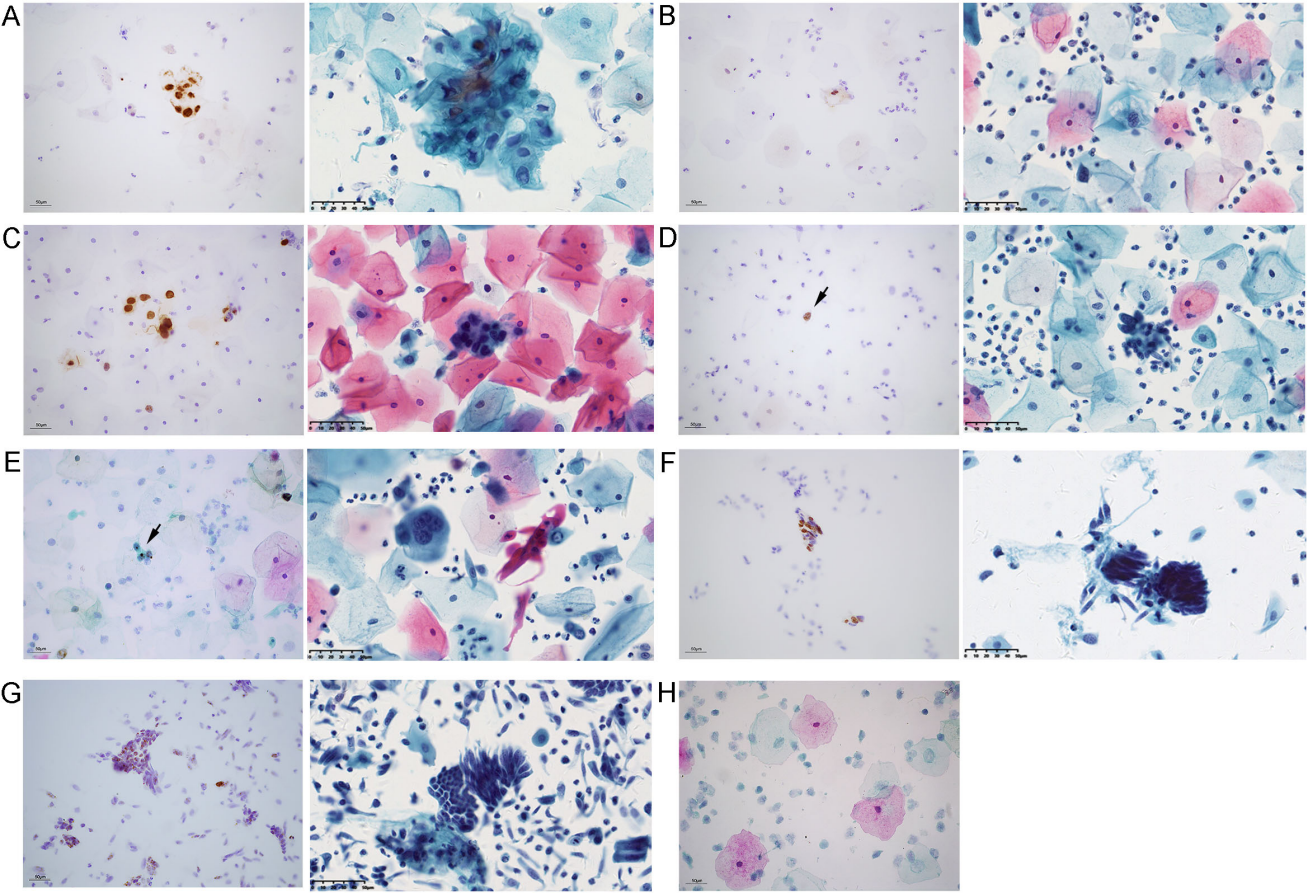
RNAscope positive signals and corresponding LBC images in different cervical lesions. Representative images of cervical lesions obtained by RNAscope (left) and corresponding LBC images (right) in each panel. **(A)** LSIL: diffuse positive signals throughout the nucleus (left); lesional cells arranged in clusters (right). **(B)** Binuclear koilocyte in LSIL. **(C)** HSIL: diffuse positive signals throughout the nucleus (left). **(D)** Positive signals in scattered abnormal cells of HSIL smear (black arrow, left panel). **(E)** SCC: distinct brown punctate nuclear signals (black arrow indicates positive signals, left panel). **(F)** Adenocarcinoma: diffuse positive signals throughout the nucleus (left). **(G)** AGC smear showing cell clusters with diffuse punctate positive signal. **(H)** Negative results in samples without epithelial lesions. Scale bar, 50 μm (applies to all panels).

At 20-40x magnification, positive signals appeared as patchy or distinct nuclear brown spots, often clustering into dense aggregates (8). Surrounding normal cells served as internal controls; absence of brown nuclear dots indicated negativity. Under a 40x high-power microscope, a light brown, diffuse, and “misty” background staining was occassionally observed, but this was considered artifactual. No false positives was identified, and samples without epithelial lesions remained negative (**Figure 2H**). Representative staining patterns of different cervical lesions are shown in **Figure 2**.

## 4 Discussion

In gynecological oncology, RNAscope technology is primarily applied to cervical lesion biopsies, postoperative paraffin-embedded cervical cancer samples, rare HPV-associated tumors of the ovaries and other sites, enabling *in situ* detection of HPV E6/E7 mRNA expression. As a crucial adjunctive diagnostic tool, it has been incorporated into guidelines and consensus documents. The 2023 and 2024 NCCN Cervical Cancer Guidelines recommend HPV testing for all cervical adenocarcinomas, with HPV *in situ* hybridization or molecular testing as preferred options (18). In clinical practice, most patients undergo early screening with LBC, yet biopsy samples are often unavailable. Appling RNAscope to exfoliated cervical cells would enable the determination of active HPV infection and viral distribution in lesioned cells. Co-staining HPV signals with cellular structures would allow simultaneous assessment of cytological morphology and HPV expression. However, there are no such assays performed successfully in patient cervical scraped cells due to easy detachment from the slide.

This study optimized technical parameters to overcome these challenges. By comparing slide types and drying conditions, we resolved the problem of cell detachment during target retrieval. To enhance signal clarity and specificity, target retrieval duration was extended, and a permeabilization step was introduced. Cervical exfoliated cells, fixed in alcohol-based preservation solution, avoided cross-linking and offered advantages over formaldehyde, including lower toxicity and suitability for large-scale use. Although target retrieval was originally developed for FFPE tissues, it improved positive signal detection in alcohol-fixed LBC samples. Previous studies also confirm its role in enhancing immunostaining of alcohol-fixed smears (19), supporting our approach. Since alcohol-fixed cells maintain relatively intact membranes, Triton X-100 was applied as a mild permeabilizing agent to dissolve membrane lipids, facilitating probe penetration into nuclei (17). To improve cellular layer identification, Pap staining was added after hematoxylin counterstaining, enabling simultaneous visualization of cellular morphology and HPV RNA signals.

Diagnostic performance was evaluated in 30 cases. Eleven were initially diagnosed as ASCUS or negative for epithelial lesions by LBC combined with HPV PCR, but histopathology revealed LSIL. Among these, RNAscope agreed with histopathology in nine cases, while false negatives may associated with smear interpretation and fluctuations in HPV L1 capsid protein loss (20). Two additional cases were PCR-positive but RNAscope negative, potentially due to transient infection, low viral replication during incubation (21), or viral copy numbers below one per epithelial cell.

In HSIL and squamous cell carcinoma samples, one HPV+/RNAscope- case likely reflected limited cell numbers relatively in residual LBC specimens. Otherwise, RNAscope demonstrated high diagnostic accuracy, with 96.07% for HSIL and 100.00% for SCC. Although the differences from PCR testing were not statistically significant, RNAscope provided valuable visualization of HPV infection within morphologically ambiguous cells, thereby aiding in the diagnosis of complex cases. It also distinguished true precancerous lesions from non-tumor cytological changes such as immature squamous metaplasia, atrophy, and reactive inflammation. This triage function is clinically important given that the 5-year risk of CIN3+ after ASCUS and LSIL is approximately 3.8% and 6.0%, respectively (22).

One adenocarcinoma sample tested HPV16+ but not RNAscope, and no tumor cells were identified on repeat RNAscope smear review. This likely reflects sampling limitations, as cervical adenocarcinoma can arise deep within the endocervical canal beyond the reach of brushes. Cytological diagnosis of glandular lesions is inherently, challenging, as inflammation and proliferative changes can mimic neoplasia (23). Large-scale studies confirm underdiagnosis: among patients with histologically confirmed adenocarcinoma *in situ* (AIS), 60% had negative cytology, though 44% were HPV-positive (24). LBC was interpreted as atypical glandular cells (AGC), while histopathology confirmed invasive cervical adenocarcinoma (**Figure 2G**). In such cases, RNAscope detection of high-risk HPV provides definitive diagnostic evidence.

For the discordant cases between RNAscope and PCR results, we speculate that the discrepancies may arise from the distinct targets of the two methods. PCR amplifies HPV L1 DNA, while RNAscope detects HPV E6/E7 mRNA at the transcriptional level. A PCR-positive, RNAscope-negative result suggests the presence of viral DNA with undetectable E6/E7 transcription, indicative of episomal viral carriage or a rare form of transcriptionally silent integration (25). Conversely, a PCR-negative, RNAscope-positive result may indicate that the virus has integrated with active E6/E7 expression, while the L1 DNA region is either partially lost, mutated, or present at levels below the PCR detection limit.

Our findings highlight the potential of RNAscope to enhance diagnostic accuracy across different sample types. In the triage of ambiguous cytological results such as ASC-US, RNAscope enables the visualization and localization of high-risk HPV transcripts while preserving morphological context. This provides a more precise molecular basis for deciding whether to refer patients for colposcopy, helping to avoid both over-treatment and missed diagnoses. In LBC specimens with adequate cellularity, HPV signals are easily visible in both squamous and glandular cervical lesions due to the contrast of staining even under low magnification. This proves particularly valuable for detecting abnormal cells with sparse distribution that might otherwise be easily missed, as RNAscope effectively highlights these scattered cells and facilitates lesion cell localization (**Figure 2D**). For challenging histological cases with ambiguous morphology, such as distinguishing HSIL from reactive epithelial changes or atrophy, RNAscope serves as a valuable adjunct. By providing highly specific viral signal labeling, it supports pathologists in rendering more objective and accurate diagnoses. This utility extends to resolving diagnostic challenges posed by hyperchromatic crowded cell groups (HCGs) in cervical cytology, which often carry a high risk of either overdiagnosis or underdiagnosis due to their densely packed, three-dimensional structures (26, 27). By demonstrating cellular morphology alongside HPV signals, RNAscope enables clear differentiation in cervical lesions, indicating its potential for effectively distinguishing between benign and malignant entities.

Overall, previous studies have shown superior specificity of RNAscope in cervical paraffin samples compared to other assays (12, 13, 21), which indicates the assay is reliable for HPV detections. HPV PCR results examine HPV DNA without the infomation of viral subcellular locations and cervical cell transforming status, which causes unnecessary referrals in women who carried no active virus. Increases in aggressive treaments can lead to increased costs and colposcopy capacity problems (28). However, RNAscope yielded positive results in HPV-related cervical lesions, but was negative when HPV titers were too low or when lesions were unrelated to HPV. It may also help differentiate AIS from endometrial polyps, endometrial cancer, or gastric-type cervical adenocarcinoma. A key limitation of RNAscope is the potential for false negatives when too few diseased cells remain in LBC specimens, particularly in cases with insufficient cellularity or poor quality. This issue is especially pronounced in advanced invasive cervical cancers, where tumors often exhibit extensive surface necrosis, inflammatory infiltration, and deep invasive growth. Such pathological features compromise specimen quality and risk target cell loss or RNA degradation, leading to false negatives inconsistent with disease severity. Consequently, RNAscope is not suitable for all cervical specimens, its use should be avoided in samples with low cellularity or abundant necrotic debris due to the high risk of uninterpretable results. However, when scattered morphologically intact tumor cells are present on the smear, RNAscope can serve as a valuable adjunct for the identification of diseased cells and refinement of cytological diagnosis.

The specificity estimate (43.7%) and NPV estimate (35.2%) are associated with low statistical power due to the small size of the denominator population. The sample size was particularly limited within the HPV-related adenocarcinoma subgroup, resulting in *post hoc* power estimates below 50%. This suggests that the wide confidence intervals for specificity and NPV are more likely attributable to the small number of negative cases and the consequent statistical instability of the estimates, rather than reflecting poor diagnostic performance. While the overall diagnostic consistency is promising, these findings are derived from a single-center cohort with relatively imprecise estimates. Therefore, multicenter validation in a larger, more diverse population is an essential prerequisite before considering broader clinical implementation. Future work will therefore require external validation, alongside ongoing efforts in long-term follow-up.

RNAscope may not be for universal screening. Although the cost per detection item of RNAscope is higher than that of HPV DNA PCR, its value as a precision tool for women with equivocal results or for clarifying difficult diagnosis may bring system level savings. By more accurately identifying high-grade lesions, it can significantly reduce unnecessary vaginal colposcopy referrals, overtreatment, and subsequent medical costs and complications, which is particularly important for low-middle income countries with limited treatment resources. RNAscope requires a laboratory similar to IHC and trained pathologists for interpretation (9). This is more widely available than molecular PCR facilities, as IHC is often established for cancer diagnosis. The total turnaround time for RNAscope detection, from preparation to interpretation, including sample transportation and reporting time, is usually 2–3 days. This is significantly longer than rapid real-time HPV DNA testing. Therefore, we do not recommend using it for primary screening. Future implementation research should focus on developing test kits tailored to specific healthcare systems.

In summary, RNAscope provides a valuable adjunct to cytology in diagnosing HPV-related cervical lesions, including LSIL, HSIL, SCC, and adenocarcinoma. Its positivity closely correlated with pathologist-confirmed diagnoses, and its compatibility with LBC slides and staining systems enables co-localization of HPV E6/E7 mRNA signals with cellular morphology. Compared with conventional HPV genotyping testing, RNAscope provides a more intuitive, morphology-linked diagnostic approach for challenging cases, with broad clinical potential and substantial translational value.

## Data Availability

The original contributions presented in the study are included in the article/supplementary material. Further inquiries can be directed to the corresponding authors.
